# Editorial: Modulation of neuroimmune systems to preserve brain function in aging and dementia

**DOI:** 10.3389/fnins.2022.995409

**Published:** 2022-08-03

**Authors:** David J. Braun, Erica M. Weekman, Marcia N. Gordon, Claudia B. Späni

**Affiliations:** ^1^Sanders-Brown Center on Aging, University of Kentucky, Lexington, KY, United States; ^2^Department of Translational Neuroscience, College of Human Medicine, Michigan State University, East Lansing, MI, United States; ^3^Institute for Regenerative Medicine (IREM), University of Zürich, Zurich, Switzerland

**Keywords:** neuroinflammation, aging, dementia, Alzheimer's disease, immune system

Neuroinflammatory mechanisms play an important role in the progression, or lack thereof, of various aging-associated dementias. Although an active area of research, the complexity of the mechanisms involved leave much work to be done. The current collection of articles explores several specific anti-inflammatory modulators, with mixed results in various models of disease associated with neuroinflammatory damage. Chesworth et al. describe the anti-inflammatory effects of the plant flavonoid Apigenin in a mouse model of chronic neuroinflammation driven by overexpression of astrocyte IL-6. While the compound worked to normalize several markers of microglial inflammation, there was no functional rescue of impaired spatial memory (Chesworth et al.). Similarly, a study by Zajac et al. examined the effect of long-term supplementation of short-chain fatty acids on microbiome and amyloid beta-associated neuropathology in the APPswe/PS1dE9 mouse model of Alzheimer's disease (AD). While they found significant alterations in microbiome, these changes were by themselves insufficient to alter neuropathological or behavioral endpoints. In contrast, treatment with Glatiramer (an FDA-approved treatment for multiple sclerosis) was able to rescue both neuroinflammatory markers as well as behavioral deficits in the combined tau and amyloid 3xTg model of AD (Dionisio-Santos et al.). Taken together, these studies indicate that while systemic administration of anti-inflammatory compounds have the potential to reduce pathology and functional deficits, the details are vital: broadly anti-inflammatory treatments are unlikely to provide much benefit unless they are targeted to key pathologies based on the disease in question. Two reviews aim to synthesize and address some of this nuance: one by Rickenbach and Gericke that describes the role of the adaptive immune response in brain aging, and another by Garland et al. that focuses on the crosstalk between astrocytes and microglia. While the appropriate targeting of neuroinflammatory mechanisms remains a promising strategy in mitigating cognitive decline in the context of aging-associated diseases such as AD, the ongoing challenge remains to better define the “appropriate” targets for each stage and disease.

## Author contributions

DB wrote the editorial. MG, EW, and CS reviewed and approved it. All authors contributed to the article and approved the submitted version.

## Conflict of interest

The authors declare that the research was conducted in the absence of any commercial or financial relationships that could be construed as a potential conflict of interest.

## Publisher's note

All claims expressed in this article are solely those of the authors and do not necessarily represent those of their affiliated organizations, or those of the publisher, the editors and the reviewers. Any product that may be evaluated in this article, or claim that may be made by its manufacturer, is not guaranteed or endorsed by the publisher.

